# Sugar Fructose Triggers Gut Dysbiosis and Metabolic Inflammation with Cardiac Arrhythmogenesis

**DOI:** 10.3390/biomedicines9070728

**Published:** 2021-06-25

**Authors:** Wan-Li Cheng, Shao-Jung Li, Ting-I Lee, Ting-Wei Lee, Cheng-Chih Chung, Yu-Hsun Kao, Yi-Jen Chen

**Affiliations:** 1 Division of Cardiovascular Surgery, Department of Surgery, Wan Fang Hospital, Taipei Medical University, Taipei 11696, Taiwan; wanlicheng80@gmail.com (W.-L.C.); leeshaojung@gmail.com (S.-J.L.); 2 Division of Cardiovascular Surgery, Department of Surgery, School of Medicine, College of Medicine, Taipei Medical University, Taipei 11031, Taiwan; 3 Cardiovascular Research Center, Wan Fang Hospital, Taipei Medical University, Taipei 11696, Taiwan; 4 Taipei Heart Institute, Taipei Medical University, Taipei 11031, Taiwan; michaelchung110@gmail.com; 5 Division of Endocrinology and Metabolism, Department of Internal Medicine, School of Medicine, College of Medicine, Taipei Medical University, Taipei 11031, Taiwan; agleems29@gmail.com (T.-I.L.); b8801138@tmu.edu.tw (T.-W.L.); 6 Division of Endocrinology and Metabolism, Department of Internal Medicine, Wan Fang Hospital, Taipei Medical University, Taipei 11696, Taiwan; 7 Department of General Medicine, School of Medicine, College of Medicine, Taipei Medical University, Taipei 11031, Taiwan; 8 Division of Cardiology, Department of Internal Medicine, School of Medicine, College of Medicine, Taipei Medical University, Taipei 11031, Taiwan; 9 Division of Cardiovascular Medicine, Department of Internal Medicine, Wan Fang Hospital, Taipei Medical University, Taipei 11696, Taiwan; 10 Graduate Institute of Clinical Medicine, College of Medicine, Taipei Medical University, Taipei 11031, Taiwan; 11 Department of Medical Education and Research, Wan Fang Hospital, Taipei Medical University, Taipei 11696, Taiwan

**Keywords:** arrhythmia, fructose, heart–gut axis, inflammation, microbiota

## Abstract

Fructose is a main dietary sugar involved in the excess sugar intake-mediated progression of cardiovascular diseases and cardiac arrhythmias. Chronic intake of fructose has been the focus on the possible contributor to the metabolic diseases and cardiac inflammation. Recently, the small intestine was identified to be a major organ in fructose metabolism. The overconsumption of fructose induces dysbiosis of the gut microbiota, which, in turn, increases intestinal permeability and activates host inflammation. Endotoxins and metabolites of the gut microbiota, such as lipopolysaccharide, trimethylamine N-oxide, and short-chain fatty acids, also influence the host inflammation and cardiac biofunctions. Thus, high-fructose diets cause heart–gut axis disorders that promote cardiac arrhythmia. Understanding how gut microbiota dysbiosis-mediated inflammation influences the pathogenesis of cardiac arrhythmia may provide mechanisms for cardiac arrhythmogenesis. This narrative review updates our current understanding of the roles of excessive intake of fructose on the heart-gut axis and proposes potential strategies for inflammation-associated cardiac vascular diseases.

## 1. Introduction

The excessive consumption of sweets is a risk factor for cardiovascular diseases (CVD), and the major chemical feature of sweets is fructose [1,2]. Moreover, high fructose intake can increase cardiovascular mortality [2]. Epidemiological studies have suggested a possible link between the intake of fructose, including high-fructose corn sugar, with CVDs [3,4,5,6]. The main source of fructose in the food comes from fructose-containing sweeteners, sucrose, and high fructose corn syrup, in sugar-sweetened beverages and food additives [7]. Intake of sugar-sweetened beverages has been consistently linked to an increased risk of obesity, type 2 diabetes, and CVDs in various populations [8]. Fructose mediates oxidative stress, inflammation, increased intestinal permeability, endothelial dysfunction, and gut microbiota dysbiosis, and it then further aggravates metabolic syndrome (MetS) by causing tissue and organ dysfunction [9]. Evidence has suggested that fructose induces systemic inflammation and activates inflammatory signaling in local tissues and organs, including the liver, kidneys, gut, and heart [10,11,12]. Additionally, excess fructose intake may be associated with risk factors for heart disease, such as non-alcoholic fatty liver disease (NAFLD), obesity, diabetes, kidney dysfunction, and dyslipidemia [13,14,15,16,17,18]. Controlled dietary intervention studies in humans have discovered that fructose intake increased the risk posed by cardiovascular risk factors, particularly increased circulating lipid level and reduced insulin sensitivity [2]. A low carbohydrate diet has been shown to improve blood lipids with an increase in high-density lipoprotein and decreases in triglycerides and small dense low density lipoprotein [19]. Moreover, low carbohydrate diet can reduce inflammation, blood pressure, and fasting blood glucose and enhance insulin sensitivity. These clinical and laboratory results indicate that fructose overconsumption plays a vital role in the pathogenesis of MetS and drives the chronic inflammation that promotes CVDs.

## 2. Fructose-Mediated Metabolic Disorders as a Driver of Cardiac Arrhythmia

Fructose has received much attention as a possible contributor to metabolic diseases [20]. The long-term intake of fructose causes dysbiosis of the microbiota, leading to an increased permeability of gut barrier, progression of MetS, chronic kidney disease (CKD), hepatic inflammation, NAFLD, and insulin resistance (IR) [21,22]. The overconsumption of fructose is considered a main factor for developing type 2 diabetes, and a high-fructose diet is largely used to induce type 2 diabetes in rodents [23,24]. NAFLD caused by excess fructose intake is attenuated by the blocking of heme oxygenase-1 (HO-1)-sirtuin1 (SIRT1) signaling in the hepatocytes of mice [25]. Clinical trials have showed that 9 days of short-term fructose restriction can reduce liver fat, visceral fat, and the conversion of fructose to fat in the liver and can improve insulin kinetics in children with obesity [26]. These findings have suggested that excess fructose intake mediates MetS-promoting and CVD-inducing by gut microbiota dysbiosis.

**Figure 1:**
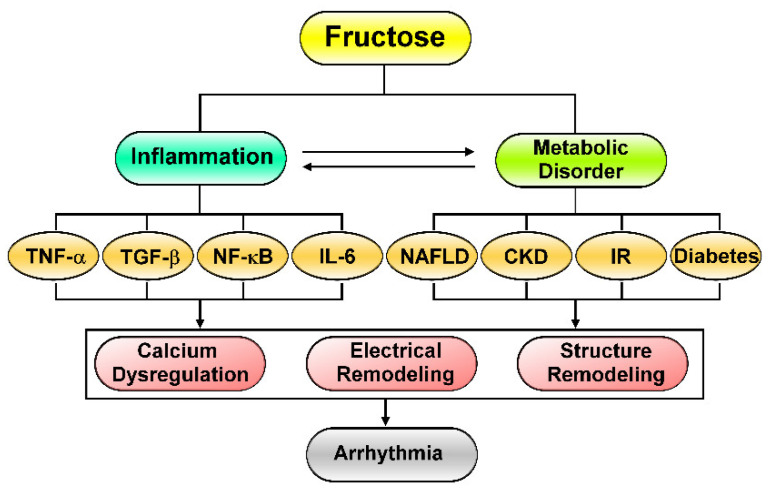
Effects of excessive fructose intake on cardiac remodeling and arrhythmia. Excessive fructose intake promotes inflammation and stimulates metabolic disorders, leading to cardiac arrhythmogenesis and aggravating its negative effects on cardiac remodeling and arrhythmia. (Left) Fructose that potentiates inflammatory signaling (TNF-α, TGF-β, NF-κB, and IL-6). (Right) Fructose that activates metabolic disease (NAFLD, CKD, IR, and diabetes). TNF-α: tumor necrosis factor alpha, TGF-β: transforming growth factor beta, NF-κB: nuclear factor kappa B, IL-6: interleukin 6, NAFLD: non-alcoholic fatty liver disease, CKD: chronic kidney disease, IR: insulin resistance.

Studies have demonstrated that the crosstalk between the gut microbiome, immune system, and liver contributes to the role of the gut–liver axis in NAFLD progression [27]. Patients with NAFLD have a greater risk of cardiac arrhythmia, including atrial fibrillation (AF) and ventricular arrhythmia [28,29]. AF is the sustained arrhythmia most associated with high mortality and morbidity [30]. A meta-analysis indicated that NAFLD is related to an increased risk of AF in older adults (particularly in patients with diabetes) [31]. Animal experiments have revealed that high-fructose diets fed to rats increased renal hypertrophy, producing chemotactic factors, such as intercellular adhesion molecule-1 and monocyte chemoattractant protein-1, that promote CKD, which is a key factor that precipitates AF genesis [32]. Calcium dysregulation may trigger AF genesis by enhancing triggered activity. Additionally, fructose-mediated IR was found to cause both calcium dyshomeostasis and atrial structural remodeling, thereby contributing to AF progression [33]. Experimental studies have verified that significant atrial remodeling and autonomic dysfunction occur in diabetes and increase susceptibility to AF development [34]. Individuals with diabetes had a higher risk of AF progression, and the risk increased with worse glycemic control and longer durations of diabetes treatment [35]. Because the overconsumption of fructose was linked to numerous metabolic and inflammation diseases, which were tightly connected to the progression of CVDs and cardiac arrhythmogenesis, fructose-mediated metabolic disorders may play a critical role in the pathogenesis of cardiac arrhythmia (Figure 1).

## 3. Effects of Excess Fructose Intake on Cardiac Arrhythmogenesis

Several animal experiments have established a causal relationship between high fructose intake and cardiac arrhythmogenesis [36]. A high-fructose diet increased the severity of ischemia-induced arrhythmias and the size of infarctions in fructose-fed rats [37]. Additionally, in rats, a high-fructose diet induced metabolic alterations, reduced myocardial total antioxidant capacity, and increased high oxidative stress [38]. Moreover, a high-fructose, high-fat diet reduced the conduction velocity of prediabetic rats [39]. Additionally, rats in the high-fructose group exhibited a decrease in left ventricular (LV) ejection fraction and an increase in LV chamber size [40]. Cardiac myocytes from mice that were fed a fructose-rich diet exhibited arrhythmias caused by the exacerbation of Ca^2+^/calmodulin-protein kinase (CaMKII) activity, phosphorylation of ryanodine receptor 2 (RyR2), and sarcoplasmic reticular Ca^2+^ leakage [41]. Rats that were fed a high-fructose diet exhibited decreased contractility and hypertrophy with enhanced CaMKII activity, with reactive oxygen species (ROS) production, with oxidized CaMKII, and with an enhanced CaMKII–RyR2 axis compared with rats fed a control diet [42]. The results in our previous study revealed that inflammation mediators can activate CaMKII/RyR2 signaling, which promotes cardiac electrical remodeling and atrial arrhythmogenesis [43]. These findings jointly suggest that a high-fructose diet may increase the risk of cardiac arrhythmia through the presence of myocardial ischemia, metabolic disorder, oxidative stress, impaired heart function with electrical and structural remodeling. However, the precise mechanisms underlying fructose-mediated cardiac disorder remain unclear and require further elucidation.

Dietary fructose can induce gut microbiota dysbiosis, which promotes inflammation and a metabolic phenotype [21]. Inflammation increases the risk of AF both by increasing arrhythmogenesis through AF triggers and by facilitating AF maintenance [44,45]. Excessive fructose intake is a key factor for cardiac inflammation in the heart–gut axis, which increases the risk of AF. As illustrated in Figure 1, these results suggest the critical role of dietary fructose in the pathogenesis of cardiac arrhythmias because metabolic disorders and inflammation-mediated cardiac remodeling are vital to arrhythmia. The novel concept of a heart–gut axis highlights the crosstalk between dietary fructose-mediated inflammation and cardiac arrhythmogenesis.

## 4. Fructose’s Effects on Cardiac Inflammation and Fibrosis

High fructose intake induced myocardial fibrosis through the activation of nuclear factor kappa B (NF-κB) and mitogen-activated protein kinase (MAPK) signaling [46]. Fructose-over consumption triggers cardiac fibrosis and an inflammatory response by activating NF-κB signaling and activating differentiation 36 (CD36)-mediated toll-like receptor (TLR) 4 (TLR4)/TLR6-IL-1R–associated kinase 4/1 (IRAK4/1) signaling, thereby leading to the activation of NLR family pyrin domain containing 3 (NLRP3) inflammasome [47]. Fructose can induce cardiac fibrosis and inflammation in vitro with higher levels of alpha smooth muscle-actin, collagen type I/II, and inflammatory cytokines in mice [48]. Elevation of NLRP3 inflammasome activity was identified in the atrial cardiomyocytes of AF patients [49]. Cardiomyocytes-specific NLRP3 knockin mice developed spontaneous premature atrial contractions and inducible AF, which was diminished by the NLRP3-specific inhibitor MCC950 [49]. Another NLRP3 inflammasome inhibitor-BAY 11-7082 also diminished the high fructose diet-induced NLRP3 inflammasome activation, resulting in suppression of caspase-1 activity and interleukin (IL)-1β and IL-18 production in kidney and liver [50]. Excessive fructose consumption demonstrably leads to high secretion of inflammatory cytokines, including transforming growth factor beta 1 (TGF-β1), tumor necrosis factor alpha (TNF-α), interleukin (IL)-1β, IL-18, and IL-6, in both the heart and in serum [51]. A high-fructose diet induces the levels of plasma insulin, blood glucose, retino-binding protein 4, soluble cluster of CD36, free fatty acids, cholesterol, triglycerides, and low-density lipoprotein cholesterol, along with the inflammatory cytokines TNF-α and IL-6 [52]. Thus, excessive fructose intake can induce the activation of NF-κB/NLRP3 signaling, and the secretion of cytokines may promote cardiac inflammation and cardiac arrhythmogenesis. Accordingly, fructose has been indicated to enhance inflammation that promotes cardiac remodeling and cardiac arrhythmias (Figure 1), and it is expected that targeting fructose-induced NF-κB/NLRP3 inflammasome activation and cytokine secretion might reduce cardiac dysfunction.

## 5. Heart–Gut Axis Regulates Fructose-Induced Cardiac Inflammation

The small intestine has been identified as a major organ for dietary fructose metabolism [53]. However, in general, the small intestine passively transmits glucose throughout the body [54]. High sugar consumption induces changes to the gut microbiota and induces metabolic disorders and obesity [55]. Low amounts of fructose (<0.5 g/kg body mass (BM)) are cleared by the small intestine; however, high amounts of fructose (≥1 g/kg BM) are digested by the microbiota and liver, and there were possibility mechanisms of enhancing fatty liver by conversion of fructose to hepatotoxic metabolites [54,56]. Healthy men who consumed up to 1.5 g/kg BW of fructose per day for 4 weeks had increased plasma triglyceride concentrations [57]. In the liver, fructose can metabolize to produce glucose, free fatty acid, triglyceride, uric acid, and advanced glycation end products (AGE), whereas targeting the AGE/RAGE pathway was considered to be a potential strategy for the treatment of CVDs [9,58]. A high-fructose diet induces dyshomeostasis of the gut microbiota and increases intestinal permeability, which precedes the development chronic inflammation and CVDs [55,59]. These laboratory results suggest that microbiota in the digestion of food ingredients and in the regulation of host metabolic functions in the gut are altered after fructose consumption. Fructose causes gut microbiota dysbiosis, which promotes inflammation-associated CVDs. Accordingly, the heart–gut axis has been suggested to be a potential therapeutic target for CVDs.

### 5.1. Microbiota Dysbiosis in Fructose-Mediated Cardiac Arrhythmia

The intestinal microbiome plays a vital role in the regulation of metabolic homeostasis of the entire body [60]. Excessive fructose intake promotes gut microbiota dysbiosis, thereby contributing to the pathogenesis of inflammatory disease [55]. When rats were fed a high-fructose diet (10.5 g/kg/day), they had increased levels of uric acid and serum inflammatory cytokines (IL-6, TNF-α, and macrophage inflammatory protein 2) but decreased anti-inflammatory cytokine (IL-10) levels [61]. Additionally, high fructose intake impairs gut microbiota homeostasis and intestinal barrier function, thereby contributing to the reduction in the expressions of tight junction proteins (occludin and zonula occludens-1) [61]. Accordingly, fructose-mediated dysbiosis alters gut permeability and promotes the release of inflammatory factors into the circulatory system, which can increase inflammation. Furthermore, gut microbiome dyshomeostasis and bacterial translocation are associated with the development of CVDs [62]. Gut microbiota dysbiosis affects the pathogenesis of several CVDs, including coronary artery disease, heart failure (HF), hypertension, and AF, which provide potential therapeutic targets for CVDs [63]. Gut epithelial dysfunction and imbalances in microbe-derived metabolites may enhance inflammation, loss of heart function, and morbidities in HF [64,65]. Patients with HF had increased intestinal permeability and bowel ischemia [66]. This caused the mucus of patients with HF to have larger concentrations of bacteria adherence than participants in the control group [66]. Comparisons between the bacteria in the feces of patients with HF revealed that the decreased Faecalibacterium prausnitzii population and increased Ruminococcus gnavus population were key features in the gut microbiota of patients with chronic HF [67]. The overgrowth of species of Streptococcus, Ruminococcus, and Enterococcus, and reductions in the levels of species of Alistipes, Oscillibacter, Faecalibacterium, and Bilophila, were detected in patients with AF [68]. Furthermore, patient groups with persistent AF lasting <12 months and persistent AF lasting >12 months shared many features of disordered gut microbiota and metabolism in common, which may happen in the early stage of the disease, whereas a prolonged persistent AF duration was connected to certain unique alterations [69]. Based on these findings, fructose-overconsumption in the heart–gut axis may play a key role in the interactions between diet and gut microbiota and lead to the genesis of CVDs and cardiac arrhythmias [70,71].

### 5.2. Effects of Microbial Metabolites on Fructose-Mediated Inflammation

**Figure 2:**
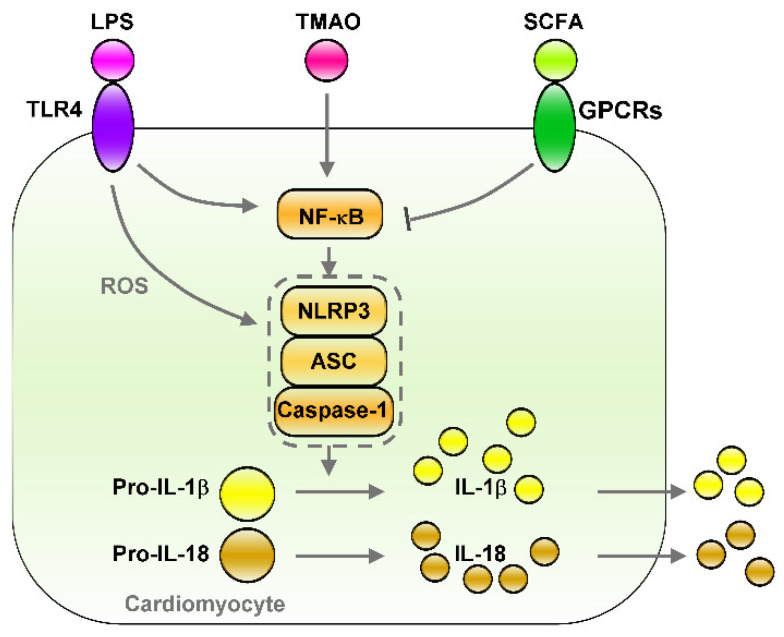
Effects of gut microbiota-derived endotoxin and metabolites on the regulation of NF-κB/NLRP3 inflammasome signaling. Gut microbiota-derived endotoxin or metabolite signaling (such as LPS/TLR4, TMAO, and SCFA/GPCRs) that altered down-stream NF-κB/NLRP3 inflammasome signaling and their effects on cardiac physiology. LPS/TLR4 and TMAO activates NF-κB/NLRP3 axis and induces secretion of IL-1β/IL-18. However, SCFA/GPCRs signaling inhibit NF-κB/NLRP3 signaling. LPS: lipopolysaccharide, TLR4: toll-like receptor 4, TMAO: trimethylamine-N-oxide, SCFA: short-chain fatty acid, GPCRs: G-protein coupled receptors, ROS: Reactive oxygen species, NLRP3: NLR family pyrin domain containing 3, ASC: apoptosis-associated speck-like protein containing a caspase recruitment domain, Pro-IL-1β: Pro-form interleukin 1 beta, Pro-IL-18: pro form interleukin 18, IL-1β: interleukin 1 beta, IL-18: interleukin 18.

The gut microbiome functions as an endocrine organ by producing metabolites that can affect the host’s health [72]. However, knowledge of the role of microbial metabolites in cardiac pathogenesis remains limited. Numerous gut metabolites that are involved in dietary metabolism have been hypothesized to be linked to pathologies, such as atherosclerosis, HF, CKD, obesity, type 2 diabetes mellitus, and AF [72,73,74,75,76]. The intestinal barrier represents a functional boundary that protects the host from leakage of the intestinal microbiota or microbial metabolites into the circulatory system [75]. Fructose mediates the loss of tight junction proteins, thereby increasing gut permeability and resulting in the translocation of bacteria and bacterial endotoxins into circulation [21]. A defective intestinal epithelial barrier, presence of dysbiosis, and enhanced translocation of gut microbial products all promote cardiac inflammation [70,71]. As summarized in Figure 2, the metabolites of microbiota may alter cardiac inflammation through NF-κB/NLRP3 signaling, including via the lipopolysaccharide (LPS)/TLR4, trimethylamine N-oxide (TMAO), and short-chain fatty acid (SCFA)/G-protein coupled receptors (GPCR) pathways.

### 5.3. The LPS–TLRs Axis Mediates Inflammation

Gut microbiota dyshomeostasis caused by high fructose intake, accompanied by increased intestinal wall permeability and LPS production, contributes to inflammation and IR [77]. The excess LPS enters circulation, proceeding to enhance inflammation by activating TLR4 signaling [78]. TLR4 is a pattern recognition receptor expressed by macrophages, endothelial cells, enterocytes, and dendritic cells (DC) [78]. The LPS–TLR4 axis mediates the induction of inflammatory cytokines, chemokines, and cell adhesion molecules [78,79,80,81]. Animal studies have suggested that fructose leads to increased intestinal permeability, causing endotoxin levels of LPS to be translocated; this activates TLR4 in liver Kupffer cells, and that activation in turn leads to the release of TNF-α [82,83,84]. Mice that were fed fructose and fat exhibited elevated TLR4 and endotoxin levels, with greater amounts of infiltrating macrophages and an increased polarization of Kupffer’s cells, all of which contributed to inflammation-associated disorders [21]. The activation of immune cells plays a key role in immunological responses and is also involved in the pathophysiological mechanisms of CVDs [85,86]. Thus, the targeting of LPS-TLR4 signaling may reduce fructose-induced cardiac inflammation.

The long-term consumption of fructose is connected with the loss of tight junction in the intestine, increased endotoxin translocation, and increased TLRs induction in the heart [47,87,88,89]. In patients with AF, serum LPS levels predicted the occurrence of major adverse cardiovascular events and were inversely associated with adherence to a Mediterranean diet (Med-diet) [71]. The administration of LPS in a canine model caused increases in the levels of TNF-α and IL-6 in circulation and the right atrium [90]. The underlying mechanism may involve the LPS-induced activation of NF-κB and an increase in both connexin 43 expression and lateral distribution through the α1-adrenergic receptor-dependent pathway that promotes AF inducibility in LPS-induced systemic inflammation [90]. Moreover, the LPS–TLR4 axis mediates NLRP3 inflammasome activation and induces the secretion of IL-1β/IL-18, which promotes cardiac inflammation [91]. Recent studies have also demonstrated that age-related microbiota dysbiosis promotes AF partially through the microbiota–gut–atria axis [92]. According to the model of fecal microbiota transplantation, aged rats treated with youthful microbiota ameliorated atrial NLRP3-inflammasome activity and the intestinal structure alternation, which inhibited the progression of aged-related AF [92]. Therefore, maintaining gut microbiota homeostasis helps prevent the activation of inflammation-inducing CVDs. Thus, gut dysbiosis induced by the LPS–TLRs axis is strongly associated with cardiac inflammation and arrhythmogenesis. Targeting of TLR4–NLRP3 inflammasome signaling could reduce fructose-induced cardiac inflammation and fibrosis [47]. As illustrated in Figure 2, LPS–TLR4 axis is involved in mediating the NLRP3 inflammasome activity that may promote cardiac arrhythmogenesis. The aforementioned studies elucidate microbiota dysbiosis-mediated cardiac inflammation and AF, thus aiding the formulation of therapeutic strategies that involve the modulation of gut microbiota composition. Correspondingly, blocking LPS/TLR4/NLRP3 signaling might lesson inflammation-related cardiac arrhythmia.

### 5.4. TMAO Induces Inflammation

Dietary fructose causes gut microbiota dysbiosis, which induces TMAO generation [93,94]. High-fructose diets also induced higher levels of TMAO in serum from mice and rats compared with those of controls [95]. Chronic high-fructose diets lead to the dyshomeostasis of gut microbiota, which diminishes gut barrier function and increases permeability, and subsequent bacterial translocation, and the gut microbe–derived trimethylamine (TMA) enters the liver through the portal vein [96]. Hepatic enzyme flavin-containing monooxygenase 3 (FMO3) can metabolize gut microbe–derived TMA to produce TMAO [97]. TMAO has also been discovered to negatively affect intracellular calcium homeostasis and cardiomyocyte contractility [98]. TMAO-treated cardiomyocytes exhibited an accumulation of glycogen and a deposition of a lipofuscin-like pigment, suggesting the occurrence of higher oxidative damage and an altered energetic metabolism of cells [98]. TMAO administration induced cardiac fibrosis in rats, and reducing TMAO synthesis with antibiotics reduced the cardiac fibrosis that had been induced by transverse aortic constriction [99]. TMAO was also discovered to promote cardiac fibroblast proliferation and collagen production in a dose-dependent manner through TGF-β/Smad3 signaling [99]. Furthermore, TMAO was observed to enhance the differentiation of fibroblasts into myofibroblasts by activating the TGF-βRI/SM2 pathway [100]. These laboratory results suggest that excessive fructose consumption may induce gut microbiota dyshomeostasis and increase TMAO accumulation in the serum, thereby promoting cardiac fibrosis.

TMAO can stimulate inflammatory pathways and NF-κB/NLRP3 signaling, thereby promoting the progression of CVDs [101,102]. One study found that the expression of NF-κB/NLRP3/caspase-1/IL-1β signaling in the aortic arteries was significantly elevated in TMAO-injected rats compared with the control group [102]. TMAO also enhanced doxorubicin-induced cardiac dysfunction and cardiac fibrosis, conditions which were indicated by increased collagen production and NLRP3 inflammasome activation [103]. Remarkably, the NLRP3 inhibitor (MCC950), a caspase-1 inhibitor (YVAD), in addition to NLRP3 short interfering RNA lessened TMAO-mediated NLRP3 inflammasome activation, then leading to inhibition of inflammation in human umbilical vein endothelial cells (HUVEC) [104]. Moreover, TMAO may have a direct influence on cardiac oxidative stress, but it stimulated mitochondrial ROS generation and inhibited manganese superoxide dismutase 2 (SOD2) activation and sirtuin 3 (SIRT3) expressions in HUVECs and aortas in apolipoprotein E (ApoE)-KO mice with atherosclerosis [104]. TMAO-induced endothelial NLRP3 inflammasome activation was also found to be ameliorated by the mitochondrial ROS scavenger Mito-TEMPO or SIRT3 overexpression in HUVECs [104]. Therefore, inhibiting NF-κB/NLRP3 signaling can help diminish TMAO-induced cardiac inflammation.

Numerous studies have identified TMAO as a potential promoter of chronic diseases containing atherosclerosis [105]. TMAO/protein kinase C (PKC)/NF-κB/vascular cell adhesion molecule (VCAM)-1 signaling can promote atherosclerosis progression by inducing endothelial dysfunction, including by decreasing endothelial self-repair and increasing monocyte adhesion [106]. TMAO-related increases in inflammatory monocytes might raise the cardiovascular risk of patients [107]. By exacerbating autonomic remodeling and increasing inflammatory cytokines in ganglionated plexuses by activating NF-κB signaling, TMAO can increase the risk of atrial arrhythmogenesis in a rapid atrial pacing–induced AF model [108]. Furthermore, TMAO binds and activates PKR-like endoplasmic reticulum stress kinase (PERK) at physiologically relevant concentrations and induces the transcription factor forkhead box protein O1 (FOXO1), which is the main driver of metabolic disease, in PERK-dependent signaling [109]. The inhibition of TMAO, either by managing the gut microbiota or by inhibiting the TMAO-synthesizing enzyme FMO3, can reduce PERK activity and FOXO1 expression levels in the liver [109]. Moreover, TMAO can activate the cardiac autonomic nervous system and increase ischemia-mediated ventricular arrhythmias, and TMAO was identified as a predictor of ischemic stroke in patients with AF and of major unfavorable cardiac events in patients with ischemic HF [110,111,112]. Increased plasma TMAO was considered to be a predictor of the risks of AF [73]. TMAO-induced NF-κB/NLRP3 signaling, which promote cardiac inflammation (Figure 2). Therefore, targeting the TMAO-mediated activation of NF-κB/NLRP3 signaling or PERK-dependent pathways may reduce TMAO-associated inflammation.

## 6. Therapeutic Strategies for Fructose-Mediated Inflammation

The targeting of inflammation can potentially reduce the risk of cardiac arrhythmia, and the blocking of gut microbiota-mediated inflammation by reducing fructose intake, inhibiting inflammation signaling, and administering probiotics and dietary SCFAs may reduce the risk of CVDs. These approaches can ground novel therapies that target inflammation-associated cardiac arrhythmias and AF.

### 6.1. Dietary Interventions

Low carbohydrate diet can help people with diabetes better manage their blood sugar levels and body weight [113]. In addition, low carbohydrate diet was shown to substantially and sustainably reduced blood pressure and body weight with marked improvement in lipid profiles of type 2 diabetes patients [114]. McKenzie et al., showed that adequate carbohydrate restriction to achieve nutritional ketosis in adults with type 2 diabetes for 10 weeks can be effective in improving glycemic control and weight loss with decreasing medication use [115]. A Med-diet can beneficially influence the gut microbiota and related metabolome. Significant associations were found between the intake of vegetable-based diets and advantageous microbiome-correlated metabolomic profiles [116]. Conversely, higher urinary TMAO levels were found in individuals with a lower adherence to the Med-diet [116]. The Med-diet supplementation may also reduce the risk of AF, including reductions in systemic oxidative stress [117,118]. Higher fiber intake reduced inflammation and lowered the risks of major adverse cardiovascular events in end-stage kidney disease patients receiving dialysis [119]. These clinical results suggest that lowering sugar consumption, eating a balanced diet, and increasing vegetable intake help people maintain good health, reduce high-fructose–mediated cardiac inflammation, and lower CVD risk.

### 6.2. Probiotics for Controlling Cardiac Inflammation

As indicated in Table 1, an increasing amount of evidence has demonstrated that probiotics benefit the cardiovascular system. Clinical trials have revealed that when compared with a placebo, probiotic consumption reduced blood pressure, total cholesterol, and triglyceride levels in patients with type 2 diabetes [120]. Additionally, the administration of the probiotic Lactobacillus *(L.) rhamnosus* GR-1 attenuated postinfarction remodeling and HF in rats through its immunomodulatory activity in the gut [121]. Moreover, the co-supplementation of Vitamin D and a probiotic (containing Bifidobacterium bifidum, *L. acidophilus*, *L. reuteri*, and *L. fermentum*) for 12 weeks in patients with diabetes with coronary heart disease improved their mental health, serum high-sensitivity C-reactive protein levels, plasma NO levels, total antioxidant capacity, glycemic control, and HDL-cholesterol levels [122]. In a rat model, probiotics (containing Bifidobacterium (B.) breve, *L. casei*, *L. bulgaricus*, and *L. acidophilus*) exhibited a cardioprotective effect on infarct-like myocardial injury by suppressing oxidative stress damage and TNF-α [123]. In another study on rats, probiotic treatment (*L. plantarum KY1032* and *L. curvatus HY7601*) lowered the levels of plasma glucose, insulin, triglyceride, and oxidative stress that had been induced by a high-fructose diet [124]. The mice with high-fat high-fructose diets and exposure to intermittent hypoxia exhibited increased levels of inflammation markers (NF-κB, TNF-α, IL-1β, and plasminogen activator inhibitor-1) and oxidative stress markers (4-Hydroxynonenal and 3-Nitrotyrosine) [125]. The *L. rhamnosus GG* and *L. rhamnosus GG* cell-free supernatant can activate nuclear factor erythroid 2-related factor 2 (NRF-2), which may preserve antioxidant levels and inhibit NF-κB activity and reduce cardiac inflammation [125]. Supplementation with probiotics, such as *L. rhamnosus LS-8* and *L. crustorum MN047*, produced an anti-obesity effect in mice that were fed a high-fat high-fructose diet by alleviating IR, decreasing the inflammatory response (the levels of TNF-α, IL-1β, and IL-6 in serum), and altering microbiota homeostasis; this, in turn, decreased the levels of circulating LPS and increased the fecal levels of SCFAs [126]. Another probiotic, *L. kefiri*, prevented the negative effects of a high-fructose diet, which were epidydimal adipose tissue expansion and reduced levels of plasma triglycerides, adipocyte secreted-leptin, and local inflammation (TNF-α, IL-1β, IL-6, and interferon gamma) in the epidydimal adipose tissue in mice [127]. Thus, probiotic treatments could suppress high-fructose-diet-induced cardiac inflammation.

### 6.3. Effects of SCFAs on Controlling Inflammation

SCFAs are the ligands for GPCRs, which contain GPR43, GPR41, and GPR109A, that trigger anti-inflammatory signaling cascades [128]. SCFAs also modulate immune responses, partially by affecting gene expressions and the epigenome through the inhibition of histone deacetylases (HDAC) [129,130]. SCFAs are saturated aliphatic organic acids that comprise one to six carbon atoms, of which propionate, acetate, and butyrate are the most abundant and are produced by anaerobic fermentation of dietary fiber in the gut [131]. Firmicutes (gram-positive) and Bacteroidetes (gram-negative) are the most abundant phyla in the intestines, with members of Firmicutes mainly producing butyrate, whereas acetate and propionate are the primary metabolic end products of members of Bacteroidetes [132]. SCFA butyrate protects intestinal epithelial cells and stabilizes hypoxia-induced factors and, thus, attenuates local and systemic inflammation [133]. Dietary-derived butyrate inhibits innate lymphoid cells and subsequently reduces lung inflammation, airway hyperreactivity, and eosinophilia in an allergic asthma murine model [134]. SCFAs can reduce impairments of the intestinal epithelial barrier due to their protection against high-fructose-diet-induced neuroinflammation [135]. Clinical studies have revealed that daily oral supplementation of 10^10^ of *Akkermansia muciniphila* bacteria (live or pasteurized) can improve insulin sensitivity and reduce insulinemia and plasma total cholesterol in overweight or obese insulin-resistant volunteers relative to a placebo. After 3 months of supplementation, *Akkermansia muciniphila* reduced the levels of the relevant blood markers for liver dysfunction and inflammation [136]. The butyrate–GPR109A axis inhibited LPS-induced NF-κB activation in colonic cell lines and in the colon of mice [137]. SCFAs, as an HDAC inhibitor, can protect the intestinal barrier from disruption by inhibiting the LPS–NLRP3 inflammasome axis [138]. Acetate diminishes NLRP3 inflammasome activation through GPR43 and Ca^2+^-dependent mechanisms, which underscores the mechanism of metabolite-attenuated NLRP3 inflammasome activity that mitigates CVD development [139]. As illustrated in Figure 2, SCFA treatment can inhibit NF-κB/NLRP3 signaling, which may prevent inflammation-associated heart arrhythmia.

The supplementation of propionate (200 mmol/L) in drinking water attenuated angiotensin II-mediated cardiac fibrosis, hypertrophy, and vascular dysfunction in both wild-type and ApoE KO mice [140]. Additionally, the administration of propionate reduced the population of splenic effector/memory T-cells and the population of splenic T helper 17 cells in both models; it also reduced local cardiac immune cell infiltration in wild-type mice [140]. Furthermore, natriuretic peptide receptor 1 (Npr1) gene-disrupted heterozygous (Npr1^+/−^, 1-copy) mice exhibited significantly increased levels of hypertrophic markers, including those of beta-myosin heavy chain (β-MHC) and proto-oncogene (c-fos and c-jun) inflammation markers (NF-κB, MMPs), compared with 2- and 3-copy mice. Npr1 1-copy mice that were treated with sodium butyrate exhibited a significantly decreased expression of hypertrophic markers and HDAC activity. Moreover, the administration of sodium butyrate significantly reduced the systolic and diastolic parameters and increased fractional shortening of Npr1^+/−^ mice hearts [141]. Butyrate, with its anti-inflammatory properties, can facilitate macrophage phenotypic homeostasis between M1 and M2 and improves myocardial infarction-induced cardiac dysfunction and sympathetic neural remodeling, thereby reducing ventricular arrhythmias [142]. Abdominal aortic banding is a frequently used model for creating LV hypertrophy and HF, and sodium butyrate inhibited partial abdominal aorta constriction (PAAC)-induced cardiac hypertrophy by reducing LV collagen levels and wall thickness through targeting class-I HDACs [143,144]. A high-fiber diet intervention and supplementation with acetate each significantly attenuated the development of cardiac hypertrophy, hypertension, and cardiorenal fibrosis in the context of mineralocorticoid excess [145]. As indicated in Table 2, SCFA administration exerts a protective effect against CVDs.

### 6.4. HDACs’ Inhibition of Cardiac Inflammation

SCFA exerts its beneficial effects by inhibiting inflammation through the activation of GPR41/43 signaling and reduction in HDAC levels [146]. HDACs play key roles in the progression of CVDs and contribute to AF generation [147]. Inhibition HDACs was recommended as a novel therapeutic strategy for cardiac arrhythmia and AF [147,148]. HDAC11 was significantly overexpressed in both human and mouse diabetic HF hearts [149]. Knockout of HDAC11 improved dyslipidemia and reduced inflammation in the heart of mice fed with fructose when compared with controls [149]. The HDAC inhibitor (HDACi) recovers cardiac function by reducing the expression of inflammatory cytokines and by ameliorating inflammatory cell infiltration in the heart [150]. Our previous laboratory results indicated that HDACi (MPT0E014 or MS-275) treatments ameliorated TNF-α-induced mitochondrial dysfunction with increased mitochondrial superoxide production and decreased ATP synthesis in atrial cardiomyocytes [151]. MPT0E014-treated pulmonary vein cardiomyocytes had reduced calcium transient amplitudes, sodium-calcium exchanger currents, and the expression of ryanodine receptor [152]. Additionally, MPT0E014-treated rabbits had less AF and shorter AF duration in AF-rabbit model (with rapid atrial pacing and acetylcholine infusion) rabbit model than controls [152]. Moreover, MS-275 ameliorated hyperglycemia, insulin resistance, TNF-α expression, and stress signaling in skeletal muscle in high fat high fructose-fed mice [153]. Therefore, HDACi treatment is a potential strategy for suppressing cardiac arrhythmogenesis.

## 7. Conclusions and Future Perspectives

**Figure 3:**
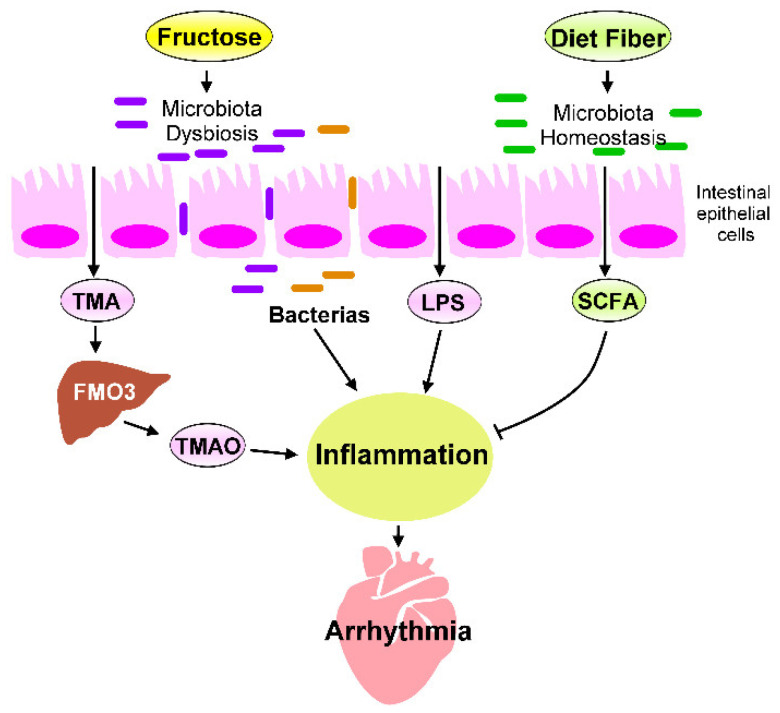
Fructose-mediated heart–gut axis disorder that promotes inflammation and cardiac arrhythmogenesis. Dietary components, such as fructose or dietary fiber, serve as crucial environmental factors that influence the homeostasis of gut microbiota and alter gut microbiota-derived metabolites. Excessive fructose intake promotes microbiota dysbiosis, which increases the production of trimethylamine (TMA), which is then converted into trimethylamine-N-oxide (TMAO) by the flavin-containing monooxygenase 3 (FMO3) expressed in the liver. SCFAs are generated through the fermentation of dietary fibers by gut microbiota. SCFAs are crucial players in regulating the beneficial effect of dietary fibers. The microbiota endotoxin and metabolites, such as lipopolysaccharide (LPS), TMAO, and SCFAs, mechanistically regulate the chronic inflammation that affects cardiac rhythm. Targeting inflammation caused by imbalanced intestinal flora may prevent cardiac arrhythmogenesis.

The heart–gut axis is a potential target for cardiovascular therapy. High-fructose diets can induce inflammation and metabolic disorders in the heart–gut axis due to cardiac arrhythmogenesis (Figure 3). Excessive fructose intake causes dysbiosis of the microbiota, which leads to increased gut barrier permeability, inflammation, progression of metabolic disease, and IR. Reducing sugar consumption and consuming dietary fiber, a Med-diet, probiotics, and SCFAs and receiving HDACi treatment can decrease high-fructose-diet-induced chronic inflammation. The targeting of excessive fructose intake-associated inflammation in the heart–gut axis can prevent cardiac arrhythmia. However, in general, studies have not determined: (1) the specific microbiota strains that can inhibit excessive fructose intake–induced heart disease; (2) how to maintain a healthy diet that avoids TMAO accumulation in the human body and how to prevent TMAO-mediated CVDs; or (3) how the SCFAs and HDACi regulate signaling that may exert an anti-inflammatory activity in the heart. Future investigations should focus on how one can maintain a balanced diet to keep their gut microbiota homeostasis and avoid systematic inflammation. Microbial population and microbiota biofunction analysis can help researchers explore the effects of probiotics on the heart–gut axis; such effects can ground promising strategies for maintaining gut microbiome homeostasis in particular and cardiac health in general. Although TMAO is a risk factor for CVD progression, TMAO mechanisms mediated CVDs, and therapeutic strategies to inhibit TMAO signaling should be explored for CVD interventions. Moreover, animal studies and clinical trials are required to analyze whether interventions that target microbiota homeostasis, inhibit TMAO signaling, or activate SCFA regulated pathways can reduce the likelihood of adverse cardiac events and prevent cardiac arrhythmogenesis. Thus, targeting the heart–gut axis may reduce the occurrence or severity of cardiac pathogenesis mediated by excess fructose consumption.

## Figures and Tables

**Table 1:** Therapeutic effects of probiotics on cardiovascular diseases.

Probiotics	Protocol	Outcomes	References
*L. rhamnosus* GR-1	Coronary artery ligation rats fed rGR-1 (10^9^ CFU/g, daily) in drinking water for 6 weeks.	Reduced cardiac hypertrophy and LV dysfunction.	[121]
*L. acidophilus*, *Bifidobacterium bifidum*, *L. reuteri*, *L. fermentum*	Patients with diabetic and coronary heart disease received vitamin D (50,000 IU) plus probiotics (8 × 10^9^ CFU, every 2 weeks) for 12 weeks.	Reduced inflammation and increased antioxidant capacity, nitric oxide, glycemic control, and high-density lipoprotein.	[122]
*B. breve*, *L. casei*, *L. bulgaricus* *L. acidophilus*	Rats fed probiotics (2 × 10^6^ CFU/mL, daily) for 2 weeks in response to isoproterenol-induced myocardial injury.	Reduced oxidative stress and inflammation and increased cardiac function.	[123]
*L. curvatus HY7601*, *L. plantarum KY1032*	Rats fed a high-fructose diet (70% *w*/*w*) for 3 weeks followed by a probiotic (10^9^–10^10^ CFU, daily) for 3 weeks.	Reduced oxidative stress, insulin resistance, and levels of plasma glucose and triglycerides.	[124]
*L. rhamnosus LS-8*, *L. crustorum MN047*	Mice fed a high-fructose high fact diet (45% kcal fat, 10% *w*/*v* fructose) and a probiotic (10^9^ CFU, daily) for 10 weeks.	Reduced insulin resistance and inflammation.	[126]
*L. kefiri*	Mice fed fructose (20% *w*/*v*) and a probiotic (10^8^ CFU, every 2 days) for 6 weeks.	Reduced adipose tissue expansion, plasma triglyceride and leptin levels, and inflammation.	[127]

*L.rhamnosus* GR-1, *Lactobacillus rhamnosus* GR-1; *B*. *breve*, *Bifidobacterium* *breve*; LV, left ventricular; CFU, colony-forming units; IU, international units.

**Table 2:** Therapeutic effects of short-chain fatty acids (SCFA) on cardiovascular diseases.

SCFAs	Study Design	Outcomes	Proposed Mechanisms	References
Propionate/Propionic Acid	Angiotensin II-treated wild-type or ApoE-KO mice fed sodium propionate (200 mmol/L, daily) in drinking water for 28–33 days.	Cardiac hypertrophy↓ Cardiac fibrosis↓ Ventricular tachyarrhythmias↓ Atherosclerotic lesion burden↓	Protects cardiac functions though regulating T helper cell homeostasis.	[140]
Butyrate/Butyric Acid	Npr1 gene-disrupted heterozygous (Npr1^+/−^, 1 copy) mice received butyric acid (0.5 mg/kg/day, daily) intraperitoneally for 14 days.	Hypertrophic markers↓ Inflammatory mediators↓ HDAC activity↓ Cardiac dysfunction↓	Suppress the cardiac expression of hypertrophic markers and proinflammatory mediators in Npr1 gene–disrupted haplotype mice.	[141]
Butyrate/Butyric Acid	Myocardial infarction rats received butyric acid (7.5 mmol/kg, daily) intraperitoneally for 3–7 days.	Cardiac dysfunction↓ Ventricular arrhythmias↓ Inflammation↓ Sympathetic neural remodeling↓	Prevents ventricular arrhythmias by inhibiting inflammation and reducing sympathetic neural remodeling.	[142]
Butyrate/Butyric Acid	PAAC-induced cardiac hypertrophy in rats fed sodium butyrate (5 mg/kg, daily) for 56 days.	LV dysfunction↓ Cardiac fibrosis↓	Prevents PAAC-induced cardiac hypertrophy through downregulation of class I HDACs.	[144]
Acetate/Acetic Acid	Mineralocorticoid-excess-treated mice fed a high-fiber diet (72.7% fiber) or acetate (200 mmol/L) in drinking water for 21 days.	Blood pressure↓ Cardiorenal fibrosis↓ Left ventricular hypertrophy↓	Prevents hypertension and cardiac fibrosis though downregulating Egr-1.	[145]

ApoE, Apolipoprotein E; KO, knockout; Npr1, natriuretic peptide receptor 1; PAAC, partial abdominal aorta constriction; LV, left ventricular; HDACs, histone deacetylases; Egr-1, early growth response protein 1.

## Data Availability

Not applicable.
